# Sympathetic dysfunction in vasovagal syncope and the postural orthostatic tachycardia syndrome

**DOI:** 10.3389/fphys.2014.00280

**Published:** 2014-07-28

**Authors:** Elisabeth Lambert, Gavin W. Lambert

**Affiliations:** ^1^Human Neurotransmitters Laboratory, Baker IDI Heart and Diabetes InstituteMelbourne, VIC, Australia; ^2^Departments of Physiology, Monash UniversityClayton, VIC, Australia; ^3^Faculty of Medicine, Nursing and Health Sciences, Monash UniversityClayton, VIC, Australia

**Keywords:** syncope, postural tachycardia, orthostatic response, sympathetic nervous activity, tilt table

## Abstract

Orthostatic intolerance is the inability to tolerate the upright posture and is relieved by recumbence. It most commonly affects young women and has a major impact on quality of life and psychosocial well-being. Several forms of orthostatic intolerance have been described. The most common one is the recurrent vasovagal syncope (VVS) phenotype which presents as a transient and abrupt loss of consciousness and postural tone that is followed by rapid recovery. Another common type of orthostatic intolerance is the postural orthostatic tachycardia syndrome (POTS) which is characterized by an excessive rise in heart rate upon standing and is associated with symptoms of presyncope such as light-headedness, fatigue, palpitations, and nausea. Maintenance of arterial pressure under condition of reduced central blood volume during the orthostasis is accomplished in large part through sympathetic efferent nerve traffic to the peripheral vasculature. Therefore sympathetic nervous system (SNS) dysfunction is high on the list of possible contributors to the pathophysiology of orthostatic intolerance. Investigations into the role of the SNS in orthostatic intolerance have yielded mixed results. This review outlines the current knowledge of the function of the SNS in both VVS and POTS.

## Introduction

Orthostatic intolerance is defined by the inability to tolerate the upright posture. It is usually associated with signs and symptoms including sensation of warmth, nausea, lightheadedness, dizziness, fatigue, abdominal pain, and visual blur. If recumbence is not achieved rapidly, syncope, defined as a temporary loss of consciousness and postural tone, can follow quickly. Syncope resolves almost immediately when the patient assumes the supine position, however after recovery, patients may complain of a “washed out” and tired feeling (Brignole et al., 2001). Syncope is an important clinical problem which accounts for approximately 1% of hospital admissions and 3% of emergency department visits with a reported mortality and major morbidity rate of over 7% (Mathias et al., 2001; Colman et al., 2004). Orthostatic intolerance is often disabling and may cause injury (Calkins and Dp, 2008). The condition represents a manifestation of many different processes and is commonly referred as a symptom, not a disease, and can be classified according to the underlying cause. The causes of syncope can be, generally, classified into six groups comprising: vascular, cardiac, neurological, psychogenic, metabolic, and syncope of unknown origin. The most common cause of syncope in the general population is the neurally mediated syncope known as neurocardiogenic syncope, vasovagal syncope (VVS) or simply “fainting” and has a lifetime cumulative prevalence of 22% (Sun et al., 2004).

Orthostatic intolerance does not always lead to syncope. Such is the case for patients with the postural tachycardia syndrome (POTS), a condition characterized by a heart rate (HR) increment of 30 beats/min or more within 10 min of standing in the absence of orthostatic hypotension. The orthostatic intolerance in these patients is typically accompanied by symptoms of cerebral hypoperfusion similar to those experienced by patients suffering recurrent VVS but syncope does not occur in many of them (Nwose et al., 2009). In fact only a third of patients with POTS have frank syncope, although daily or almost daily pre-syncope occurs (Nwazue and Raj, 2013). Most patients complain of significant exercise intolerance and extreme fatigue, even to activities of daily living. Some patients with POTS also meet diagnostic criteria for chronic fatigue syndrome (Okamoto et al., 2012).

Maintenance of appropriate blood flow to critical organs on assumption of upright posture is a major physiological challenge. This challenge involves a fine balance between neural, hormonal, and hemodynamic alterations to maintain adequate arterial pressures. Sympathetic nervous system activity represents a fast-acting regulatory system that allows adaptation of the cardiovascular system during changes in posture. The activity of the sympathetic nervous system (SNS) is primarily regulated by mechanoreceptors, sensing changes in pressure and, to a smaller degree, by chemoreceptors. The arterial baroreceptors (high pressure receptors) are located in the carotid sinus and the aortic arch, and cardiopulmonary baroreceptors (low pressure receptors) are located in the great veins, atria (A- and B-receptors, Bainbridge reflex), and ventricles (Bezold-Jarisch reflex). These mechanoreceptors are stretch-activated ion channels, which influence sympathetic outflow (Chapleau et al., 1995). The carotid sinus and aortic depressor nerves convey primary baroreceptor afferent information to regions of the nucleus of the solitary tract (for review, see Vasquez et al., 1997).

In humans, normal baroreflex physiology in response to decreased venous return during upright posture involves, increased vascular sympathetic nerve activity, and decrease in parasympathetic activity, resulting in an increase in heart rate and contractility, and an increase in peripheral vasoconstriction (Rowell, 1993). These normal responses result in maintenance of arterial pressure during upright posture and protection of cerebral perfusion thereby preventing symptoms such as those reported by patients suffering from recurrent syncope or in those with POTS.

Given the importance of sympathetic nervous regulation in response to changes in posture, the cause of orthostatic intolerance has been thought to involve various degrees of sympathetic dysfunction. As the SNS is central to the neurocirculatory response to posture, it has been assumed that these neural mechanisms fail in orthostatic intolerance. This review will focus on the current knowledge of sympathetic dysfunction, in two common types of orthostatic intolerance: VVS and the POTS.

## Neuro-hormonal changes on standing

**Figure 1:**
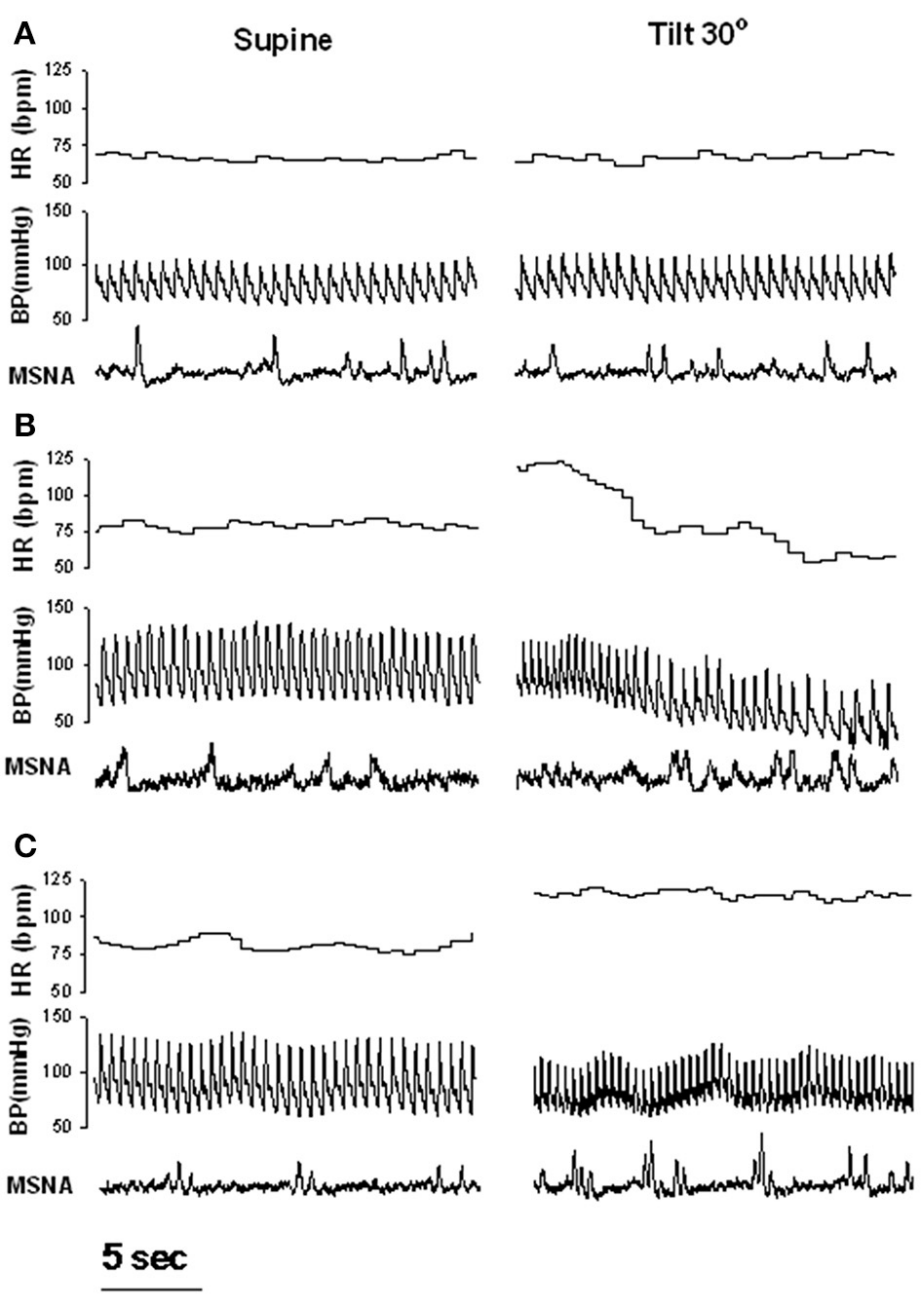
**Examples of heart rate (HR), blood pressure (BP) and muscle sympathetic nerve activity (MSNA) during supine rest and 30 degree tilt in one healthy control (A), one patient with recurrent vasovagal syncope (B), and one patient with postural tachycardia syndrome (C)**. Adapted from Vaddadi et al. (2007).

Changing posture from supine to standing leads to a rapid pooling of 300 to 800 ml of blood in the lower extremities and to the pelvic region causing thoracic hypovolemia due to an abrupt drop in venous return to the heart hence decreasing the ventricular preload. This fall in preload leads to a decrease in cardiac output (CO) and to less distention of the aortic arch and carotid sinus baroreceptors and subsequently reduced afferent baroreflex traffic to the brainstem. The unloading of the baroreceptors triggers reciprocal changes in autonomic activity with parasympathetic inhibition and sympathetic activation resulting in an increase in both HR and total peripheral resistance in order to minimize orthostatic reduction in blood pressure (BP). Increased fluid absorption has also been described in response to the increases in peripheral vascular resistance (Hinghofer-Szalkay et al., 1992). The cardiopulmonary baroreflexes are simultaneously unloaded to potentiate the actions of the arterial reflexes (Victor and Mark, 1985). Normally, orthostatic stress evokes compensatory vasoconstriction across multiple vascular beds including the skeletal muscle, which can be recorded as muscle sympathetic nerve activity (MSNA) in humans (Figure 1A). In response to graded tilt, MSNA of healthy individuals increases and correlates with the degree of the tilt angle (Mosqueda-Garcia et al., 1997). Jacob et al. (1998) studied the neurohumoral changes in response to standing in healthy individuals and noted a rapid and sustained increase in noradrenaline spillover, most likely as a result of both increased nerve firing and a reduction in norepinephrine plasma clearance (Meredith et al., 1992). This surge in noradrenaline preferentially binds α_1_-adrenoceptors to cause smooth muscle contraction and vasoconstriction. In parallel, a transient rise in plasma concentrations of adrenaline, steady elevation of plasma aldosterone concentration as well as an increase in the renin angiotensin system activity were noted. These responses were found to correlate to the physiological orthostatic hypovolemia indicated by a decrease in plasma volume reaching 13% after 14 min of standing. The hypovolemia is driven by the intravascular shift of fluid derived from the central compartment to the lower extremities and the pelvic region. The BP in the upright posture reached a nadir soon after the HR and plasma adrenaline peak. Plasma arginine vasopressin level rose with a time course similar to that of plasma renin activity. The rise in plasma renin activity and aldosterone release could exert numerous effects to maintain posture: increasing tubular Na^+^ reabsorption at the level of the kidney, direct vasoconstriction at the level of the vascular smooth muscle, and enhancement of noradrenaline release by actions on the presynaptic noradrenergic neurons and possibly also at the level of the central nervous system (Jacob et al., 1998). In addition, it is recognized that adrenaline can exert significant effects, particularly on α2- and β 2-adrenoreceptors (humoral receptors) in the circulation, while also having effects on α1- and β1-adrenoreceptors (neuronal receptors) (Hoffman et al., 1995). The strong correlation between the HR and adrenaline level described by Jacob et al. (1998) suggests that adrenaline may be an important modulator of HR change in the first few minutes of standing.

## The assessment of pre-syncope and syncope

VVS is not generally associated with cardiovascular, neurological, or other diseases, and, therefore, represents an isolated manifestation. It is usually mediated by emotional or orthostatic stress. It is diagnosed when precipitating events such as fear, severe pain, emotional distress, instrumentation, or prolonged standing are associated with typical prodromal symptoms (Alboni et al., 2004). Classical VVS generally starts at a young age, the most common age at which it first presents is 13 years (Sheldon et al., 2006). The overall incidence of syncope in individuals aged 30–62 years over a 26-year period was reported to be 3.3%. (Savage et al., 1985). The incidence is thought to increase with age, and in elderly patients in long-term nursing facilities it is approximately 6% (Lipsitz et al., 1986). In 30% of patients syncopal episodes are recurrent (Lipsitz et al., 1986).

Syncope is driven by an acute fall in mean BP to less than 40 mmHg that causes a transient interruption of cerebral blood flow for more than 8 s (Manolis et al., 1990). VVS results from excessive arteriolar dilation and inappropriate bradycardia. The sequence of events occurring during VVS is still not very well understood despite the many investigations that have been conducted (Kaufmann, 1995). The assessment of the underlying pathophysiological mechanisms leading to VVS has been difficult to determine because syncope may occur randomly and patients are generally otherwise healthy and as there are no convincing reports on reflex syncope in animals (Van Dijk, 2003), there is no perfect animal model for reflex syncope. The distinction between VVS and other causes of fainting is however essential, as the prognosis and treatment are different. The use of tilt table testing has facilitated the evaluation of the possible mechanisms underlying the differing forms of orthostatic intolerance. Tilt table testing induces orthostatic stress, resulting in maximal venous pooling, central hypovolaemia, and provocation of vasovagal syncope. The caveats of this test include limited activation of the muscle pump in the lower limbs and the fact that it is not uncommon for non-orthostatically intolerant patients to return positive results. Nevertheless the test is generally accepted to reproduce vasovagal syncope in the laboratory setting (Parry and Kenny, 1999). Lower body negative pressure (LBNP) has also been useful in identifying possible physiological mechanisms associated with syncope (Bechir et al., 2003) although it is recognized to more closely simulate hemorrhage (Convertino, 2001). Lately, it was argued that combining passive tilt testing with LBNP provided the best approach for diagnosis for VVS (Protheroe et al., 2013); this combined method is used in a few laboratories (Patwardhan et al., 2001; Batzel et al., 2009). The understanding of the pathophysiology underlying VVS is further complicated by the fact that many mechanistic evaluations have been conducted in healthy individuals who develop syncopal episodes exclusively during a tilt test or LBNP. This is an important consideration given that well-defined differences in hemodynamic characteristics between patients with recurrent syncope and healthy subjects who faint only when tilted have been established (Mosqueda-Garcia et al., 1997). Julu et al. (2003) described in ten patients the cardiovascular events leading up to pre-syncope. They showed that progressive orthostatic stress triggers a succession of BP and HR reactions that are independent of the subject's tolerance. These reactions can be categorized into four response phases including (1) full compensation, (2) tachycardia, (3) BP instability, and (4) pre-syncope. Although the mechanisms responsible for the development of syncope were uncertain, the authors postulated that it may result from an increase in sensitivity and resetting of the arterial baroreceptors functions.

## Paradoxical compensatory reflex as a cause of VVS

The ventricular theory postulates that baroreceptors react to a decrease in BP by sympathetic nervous activation leading to greater cardiac inotropy, chronotropy, and peripheral vasoconstriction (Thoren, 1979). It has first been proposed that vigorous contractions of a volume-depleted ventricle leads to activation of the cardiac C fibers (non-myelinated fibers, found in the atria, ventricles and pulmonary artery). Stimulation of these afferent C fibers leads to a “paradoxical” withdrawal of peripheral sympathetic tone and an increase in vagal tone, which, in turn causes vasodilation and bradycardia (Kaufmann, 1995). Contrary to this view, by using echocardiographic measurements of left cardiac chamber size and stroke volume, Novak and colleagues found no evidence of progressive cardiac emptying before the onset of syncope (Novak et al., 1996). Furthermore, assessment of left ventricular wall stress and segmental wall thickening preceding syncope showed no evidence compatible with activation of left ventricular mechanoreceptors (Liu et al., 2000).

## Sympathetic response to orthostatic stress and sympathetic withdrawal during VVS

Earlier studies have suggested that the vasodilation seen during VVS results from a withdrawal of sympathetic tone (Wallin and Sundlof, 1982; Van Lieshout et al., 1991). To assess this issue, direct measurements of sympathetic activity have been performed in patients using either direct recording of MSNA or noradrenaline isotope dilution methodology (spillover). Microneurography is highly reproducible and minimally invasive compared with the noradrenaline spillover technique, and offers the advantage that changes in sympathetic activity, BP and HR can be observed continuously, permitting the temporal association between variables to be evaluated. However, recording sites are often compromised when loss of postural tone occurs (Vaddadi et al., 2010). In earlier studies, since a progressive or abrupt withdrawal of MSNA before vasovagal syncope was observed (Morillo et al., 1997; Jardine et al., 1998), it was assumed that reduced sympathetic activity might play a causal role in orthostatic intolerance. In the study from Jardine et al. (1998), the fall in MSNA during presyncope correlated directly with mean BP, whereas parasympathetic activity (as measured by spectral analysis of heart rate variability) remained below baseline, suggesting that sympathetic control of total peripheral resistance was the predominant mechanism of vasovagal syncope. Muscle sympathetic nerve responses during progressive head up tilt have been examined by several investigators and results vary. Mosqueda-Garcia et al. (1997) found that the initial sympathetic response to change in circulating volume is different between patients and controls, as they noted that the MSNA increase in patients was severely reduced. Patients also exhibited a reduction in maximal increase in MSNA and plasma noradrenaline responses that were inadequate to compensate for the significant fall in arterial BP. These response were also followed by progressive MSNA inhibition followed by nerve silencing and subsequent syncope. In these patients, significant reductions in baroreflex responses were observed in the supine position as assessed with phenylephrine and sodium nitroprusside infusions. Decreased baroreflex function was therefore proposed to explain the inability of these patients to increase sympathetic outflow in response to reductions in pressure. These results are in line with those from Bechir et al. (2003) who also reported an impaired MSNA in response to LBNP in patients with VVS. These patients also displayed blunted baroreflex function but were however characterized by elevated baseline MSNA. Elevated baseline sympathetic modulation of vasomotor tone in these patients may exhaust their reserve to increase sympathetic modulation of vasomotor tone and peripheral resistance any further during an orhtostatic challenge (Sneddon et al., 1993). During routine clinical autonomic testing we observed that of the 10 patients where a MSNA recording site was maintained, only 1 patient demonstrated an abrupt cessation of MSNA at the onset of haemodynamic compromise (Vaddadi et al., 2010). All other subjects demonstrated preserved MSNA, an observation that was entirely unexpected (Figure 1B). This study challenged the notion that near-total sympathetic withdrawal, as defined by cessation of MSNA, is the final common trigger that results in hypotension during VVS. This finding of persistent nerve firing during the syncopal event in all but 1 of 10 patients is supported by observations of Cooke et al. (2009) who applied LBNP to healthy individuals to simulate hemorrhage and noted that MSNA was maintained throughout cardiovascular collapse in 40% of their subjects.

## Sympathetic nerve proteins deficiencies in VVS

**Figure 2:**
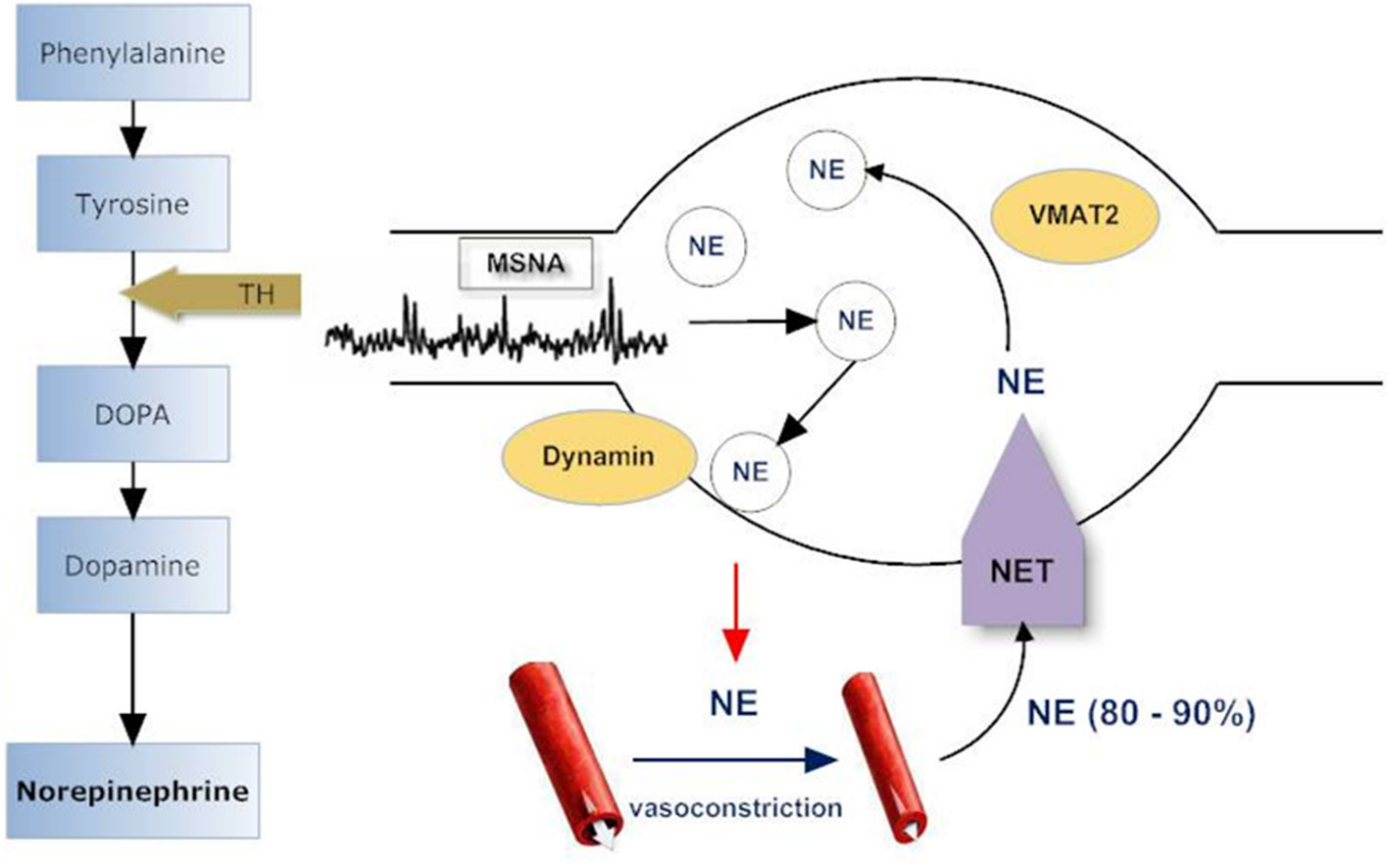
**Sympathetic nerve varicosity**. Tyrosine hydroxylase (TH) catalyzes the rate limiting step in norepinephrine (NE) synthesis. NE is stored in vesicles within the sympathetic varicosity and released to effector sites such as arterioles and venules in response to muscle sympathetic nerve firing (MSNA). Norepinephrine transporter (NET) recaptures 60–95% of released NE, and, of this, 70–90% is returned to intraneuronal vesicular storage. Vesicular monoamine transporter (VMAT2) is responsible for translocating NE from the cytoplasm into storage vesicles and is specific for sympathetic nerves. Dynamins are ubiquitous GTPases that support vesicular budding and fusion. Adapted from Vaddadi et al. (2011).

Given that patients with VVS have been reported to present two clinical phenotypes: normal systolic BP (>100 mmHg) and low systolic BP (70–100 mmHg) (Mathias et al., 2001), we compared sympathetic responses in these two distinctive groups and noticed that among patients with recurrent VVS, those with normal BP had normal MSNA at supine rest and displayed a normal MSNA response to tilt while those with low BP had an exaggerated MSNA response to tilt (Vaddadi et al., 2011). In addition, low BP patients presented with low baseline whole body noradrenaline spillover to plasma. Both patient groups had a severely blunted noradrenaline spillover response to head up tilt, with no increase in noradrenaline spillover to plasma indicating a failure of SNS response to postural change. In those with low BP, noradrenaline spillover was significantly lower at all tilt angles compared with healthy subjects and in those patients with normal BP. The finding of low noradrenaline spillover in low BP subjects contrasts with their marked increase in MSNA indicating a “mismatch” between nerve firing and noradrenaline release. A similar mismatch was present in patients with normal BP who had a normal increase in MSNA during HUT, but no increase in noradrenaline spillover. This “mismatch” between nerve firing and noradrenaline release was further investigated with analysis of sympathetic nerve proteins involved in noradrenaline synthesis, storage, release, and reuptake (see Figure 2). Proteins were harvested from subcutaneous vein biopsies obtained from the forearm in patients on a separate day of the main investigation (Vaddadi et al., 2011). Quantification of the key regulatory proteins tyrosine hydroxylase (TH), the rate-limiting enzyme in noradrenaline synthesis, and the noradrenaline transporter (NET), revealed that patients with low BP had a reduced level of both TH and NET while those with normal BP had increased NET expression. The origin of aberrant sympathetic nerve protein expression is unknown; however the nerve protein profile is likely to contribute to the abnormal response during head up tilt with subsequent orthostatic intolerance and syncope. The pathogenesis of VVS may lie at the level of sympathetic nerve proteins which regulate neurotransmitter disposition. The NET is responsible for clearing noradrenaline from the sympathetic nerve synaptic space, thus terminating the neural signal. Increased expression of NET may clear noradrenaline more rapidly, reducing neural compensatory vasoconstriction, and so predisposing to postural hypotension.

## Cardiac output

An alternative hypothesis is that the cardiac output (CO) fall is the predominant physiological event that determines hypotension. In a study of 56 patients presenting with suspected VVS who underwent head up tilt, stroke volume was computed from pressure pulsation data (Verheyden et al., 2008). There was a 50% reduction in calculated CO while systemic vascular resistance was maintained until pre-syncope. There occurred no difference in BP and HR response between VVS that was provoked by sublingual glyceryl trinitrate compared to those with a non-provoked VVS. On the other hand, an earlier study (Stevens, 1966) showed no exaggerated diminution in CO during early tilt in subjects who developed syncope between 10 and 18 min later. Jardine et al. (2002) noticed that although CO fell in all subjects during the first minute of tilt, the patients who became hypotensive were characterized by a further accelerated decline in CO, which began some minutes before syncope. Patients who developed syncope failed to maintain CO despite similar left ventricular filling pressures and lower total peripheral resistance. Jardine et al. (2002) postulated that partial MSNA withdrawal mediates venodilation resulting in decreased CO and mild hypotension early in the vasovagal reaction, whereas total MSNA withdrawal mediates arteriolar vasodilation, resulting in severe hypotension and syncope. Low CO may play an important role in the development of some episodes of VVS, but its position as the prime mover in this condition remains controversial. While studying healthy individuals developing syncope during a tilt test, Fu et al. (2012) noticed that the CO response varied before the onset of syncope. These authors observed a moderate fall in CO with coincident vasodilatation in the majority (64%) of the presyncopal subjects and a marked fall in CO, driven predominantly by a decrease in HR, with no changes in total peripheral resistance at presyncope occurring in a smaller (36%) subset. In parallel, when MSNA withdrawal occurred, it was a late event, observed after the onset of hypotension. In addition, most likely because subjects were healthy, sympathetic vasoconstriction and baroreflex sensitivity during symptom-free tilting were well preserved and, thus, an intrinsic impairment of sympathetic neural control and vasomotor responsiveness was suggested not to be the cause of neurally medicated (pre)syncope in this population. Instead, this study pointed to the fact that presyncope and syncope may be driven by a steep fall in CO.

## Structural changes in the central nervous system

Using magnetic resonance imaging voxel-based morphometry analysis, one recent study indicated a reduction of medullary volume in patients with VVS, encompassing the nucleus of the solitary tract and the caudal midline medulla, two regions of particular interest as pathological disturbance of the structural integrity of these nuclei could potentially affect autonomic function (Beacher et al., 2009). More recently, using the same approach, this finding was not replicated, but patients with VVS presented with regional atrophy in the right insular cortex as well as a significant reduction of whole right insular volume compared with controls (Kim et al., 2014). Since the insular cortex is recognized as playing a crucial role in cardiovascular modulation in particular parasympathetic autonomic tone and BP fluctuations (Nagai et al., 2010), such a defect was suggested to be associated with a decrease in sympathetic activity and a reciprocal increase in parasympathetic activity, resulting in cerebral hypoperfusion and syncope. Although results are at variance, these studies suggested that VVS may be associated with structural changes of brain nuclei controlling autonomic regulation, which predisposes the patient to abnormal cardiovascular homeostasis and recurrent syncope. Whether possible structural changes are an actual cause or consequence of repeated bouts of cerebral hypoperfusion associated with syncope remains to be determined.

## The assessment of the postural orthostatic tachycardia syndrome (POTS)

Clinical observations show that individuals with POTS represent a highly heterogenous group of patients (Benarroch, 2012). Earlier studies suggested that approximately one-half of patients have an antecedent, presumed viral illness (Schondorf and Low, 1993), although recent experience suggests that this is less common. Typical symptoms of complaints from POTS patients may include headache, poor concentration, dizziness, fatigue, tremor, chest discomfort, and shortness of breath. Many patients with POTS report a cyclical nature of their symptoms but, of note, also report symptoms not attributable to orthostatic intolerance, including those of functional gastrointestinal or bladder disorders, chronic headache, fibromyalgia, and sleep disturbances. In many of these cases, cognitive and behavioral factors, somatic hypervigilance associated with anxiety, depression, and behavioral amplification may contribute to symptom chronicity (Benarroch, 2012). While the diagnostic criteria focus on the abnormal HR increase upon standing, POTS usually presents with symptoms much more complex than a simple, albeit exaggerated, increase in HR. It is fairly common for POTS patients to have a noticeable drop in BP upon standing, but some POTS patients have no change or even an increase in BP upon standing (Grubb, 2008). Given the heterogeneity of this condition, understanding the underlying mechanisms of the tachycardia has proven challenging. Abnormal sympathetic nervous activity or reactivity may be central to the condition; however there is still no definite answer as to whether the excessive tachycardia with orthostasis in POTS patients is the cause or a consequence of sympathetic nervous dysfunction.

## Sympathetic denervation may be associated with POTS

There is evidence that some patients with POTS present with peripheral sympathetic denervation in the lower limbs. This is characterized by blunted noradrenaline release in the lower limb in response to orthostatic stress and loss of sweating in the feet on thermoregulatory sweat tests and quantitative sudomotor axon reflex testing (Jacob et al., 1999). Examination of medical records of patients with POTS seen at the Mayo Clinic in Rochester indicated that half of the patients had evidence of peripheral sudomotor denervation as estimated by abnormal sudomotor axon reflex test and thermoregulatory sweat test (Thieben et al., 2007). The primary pathophysiologic mechanism of postural intolerance in this subgroup of patients is presumed to be impaired peripheral vasoconstriction, leading to venous pooling in the lower limbs. Consistent with this possibility, a subgroup of patients with POTS have impaired indirect measures of sympathetic vasoconstriction as assessed from the BP profile during the Valsalva maneuver or head up tilt (Thieben et al., 2007). It was also noted that in some cases of POTS there was a defective vasoconstriction that produces resting venous hypertension and venous congestion, but it was not associated with abnormalities in the volume-pressure relations or overall venous capacitance while supine or during orthostasis (Stewart, 2002). This neuropathic subgroup, termed “high-flow” POTS, was characterized by reduced total peripheral resistance while supine and venous pooling in the legs on standing (Medow and Stewart, 2007). More recent investigations have focused on possible aberrant cardiac sympathetic innervation. Reduced myocardial meta-iodobenzylguanidine uptake indicating reduced cardiac sympathetic neuronal uptake function has been reported in 20% of patients with POTS, suggesting that cardiac sympathetic denervation may be present in this condition (Haensch et al., 2010). More recently it was also demonstrated that reduced myocardial meta-iodobenzylguanidine uptake correlated with the density of sympathetic and somatic C-fiber innervation measured from skin biopsies and suggested that loss of distal sympathetic innervation may produce an exaggerated sympathetic response in innervated tissues such as the heart, resulting in tachycardia (Haensch et al., 2014).

## Sympathetic overactivity may be associated with POTS

Some investigations (Streeten et al., 1988; Schondorf and Low, 1993; Furlan et al., 1998; Jacob et al., 1999), but not all (Jacob et al., 2000; Lambert et al., 2008), have reported that POTS patients have greater systemic plasma noradrenaline concentrations at rest or during orthostasis compared with healthy controls, suggesting that POTS is associated with a hyperadrenergic state. More robust and precise assessment of sympathetic activity by direct measurements of total body and regional noradrenaline spillover have been performed in a few investigations and the results have been variable. Evidence of sympathoexitation was confirmed in one study (Jordan et al., 2002), but not in others (Jacob et al., 1999; Goldstein et al., 2002a; Lambert et al., 2008). The possible hyperadrenergic features of POTS may not be simply due to increased central sympathetic outflow and noradrenaline release but potentially from an augmentation of the sympathetic signal due to enhanced receptor sensitivity or to a reduction in noradrenaline clearance during standing. One study found no evidence for a substantial increase in the sensitivity to adrenoreceptor stimulation during complete ganglionic blockade with trimethaphan indicating that the hyperadrenergic features of POTS could not be completely explained by systemic hypersensitivity of postsynaptic α1- and β-adrenoreceptors (Jordan et al., 2002). On the other hand, decreased noradrenaline clearance with standing was observed in POTS patients, suggesting that much of the increase in plasma noradrenaline was due to a decrease in clearance (Jacob et al., 1999), although reduced noradrenaline plasma clearance during head up tilt testing is not uncommon (Meredith et al., 1992). In addition, in the investigation by Jacob and colleagues, patients were hypersensitive to the heart rate-increasing effect of bolus injections of isoproterenol suggesting apparent β-adrenergic receptor hypersensitivity. The only study investigating cardiac sympathetic activity using measures of cardiac noradrenaline spillover to plasma indicated elevated cardiac sympathetic drive in patients with POTS (Goldstein et al., 2002a). The tachycardia in the upright position of POTS therefore may partly reflect increased noradrenaline delivery to adrenoceptors on myocardial pacemaker cells combined with a reduced synaptic clearance by NET, thereby potentiating the sympathetic neural signal.

The inconsistency of the results may reflect the highly recognized heterogeneity of patients with POTS (Benarroch, 2012). When assessed using MSNA, sympathetic tone has also been shown to be highly variable in POTS patients. For example one study reported that patients with POTS had increased resting MSNA, decreased responsiveness to head up tilt and similar supine MSNA baroreflex gain values (via vasoactive drug infusions) compared with healthy control subjects (Furlan et al., 1998). Contrasting results were found by Muenter Swift et al. (2005) who found that POTS patients had exaggerated MSNA responses to baroreflex challenges compared with control subjects, although resting supine MSNA values did not differ between the groups. By combining MSNA and total body noradrenaline spillover we found no evidence of sympathetic activation in POTS patients in the supine position as both resting MSNA and total body noradrenaline spillover were similar to that of controls (Lambert et al., 2008). However, an excessive nerve firing response was observed during tilt (Figure 1C) but this was unexpectedly not matched by enhanced noradrenaline spillover.

## Altered NET expression in POTS

Neuronal reuptake of noradrenaline via the cell membrane NET (uptake-1) is the main means of inactivation of noradrenaline in the heart and a key factor in regulation of delivery of noradrenaline to myocardial adrenoceptors (Eisenhofer et al., 1991). Jordan et al. (2002) first noticed that POTS patients had reduced plasma 3,4-dihdroxyphenylglycol (DHPG)/ noradrenaline ratios. Although the majority of DHPG in plasma is derived from metabolism of noradrenaline that leaks from storage vesicles (Eisenhofer et al., 2004) given that DHPG is produced intraneuronally via the action of monoamine oxidase this measure has been used as an indicator of the efficiency of the neuronal uptake of noradrenaline (Goldstein et al., 1988). Reinforcing the possibility that a defect in NET may be involved in the pathophysiology of POTS, selective NET blockade in healthy individuals creates a phenotype that resembles POTS (Schroeder et al., 2002). The first indication that altered NET activity was implicated in the hyperadrenergic state observed in POTS came from the study of a 33-year-old female with a 20-year history of POTS (Shannon et al., 2000). In response to upright posture, she experienced a 4-fold increase in plasma noradrenaline, but only a doubling of muscle sympathetic nerve activity, indicating an electrochemical dissociation in the sympathetic neuron. A point mutation in the coding region of the NET gene (*SLC6A2*) was identified that encoded a dysfunctional protein with dramatically reduced noradrenaline reuptake compared to wild-type NET. While neither this mutation, nor single nucleotide polymorphisms (SNPs) in the NET gene have been found in other unrelated POTS patients (Ivancsits et al., 2003), we have found that some POTS patients have decreased NET protein expression when compared with healthy subjects (Lambert et al., 2008). Previous work from our group has noted the possible importance of epigenetic mechanisms in NET regulation. While an early report suggested that hypermethylation of the NET gene (*SLC6a2*) may lead to transcriptional repression of *SLC6a2* (Esler et al., 2006), recent reports using more specific and robust methodology have found no evidence of methylation of the promoter of the NET gene (Bayles et al., 2013). Although methyl CpG binding protein (MeCP2) induced transcriptional repression of genes was originally proposed to be dependent on DNA methylation, recent studies, including our own, have established that this is not always the case (Bayles et al., 2010, 2012). Indeed, the expression of NET may be regulated independently of promoter DNA methylation, by mechanisms involving the binding of the transcription repressor MeCP2 and modification of histone tails (Bayles et al., 2010, 2012). Taken together, while these results do point to some involvement of NET in the pathophysiology of POTS data from Goldstein et al. (2002b) indicated that, at least in the supine position, cardiac extraction of ^3^H-labeled noradrenaline was not reduced, which suggests that high cardiac noradrenaline spillover was more likely due to increased cardiac sympathetic firing and high noradrenaline release rather than decreased noradrenaline reuptake. While evidence of reduced whole body noradrenaline plasma clearance are evident during head up tilt, whether changes in cardiac NET function are observed during orthostatic challenge remains to be determined.

## Conclusion

Both VVS and POTS are important clinical problems and although often generally considered benign conditions, frequent and recurrent episodes of syncope or presyncope can negatively affect quality of life and are associated with substantial morbidity. Understanding the pathophysiology of these conditions has proven difficult. VVS may result from withdrawal of sympathetic tone, baroreflex failure, a fall in the CO or a combination of both. POTS on the other hand may involve sympathetic denervation in the lower limbs, a hyperadrenergic state and/or abnormalities in the NET. While sympathetic dysfunction may be center to both conditions, results from studies are divergent which most likely reflects the pathophysiologic heterogeneity of orthostatic intolerance and the presence of multiple comorbidities not directly related to the actual orthostatic stress. It is therefore not surprising that patients with orthostatic intolerance pose a particular challenge in terms of management. Various drugs acting on sympathetic activity have been used. For example, β-blockers are proposed to reduce the degree of mechanoreceptor activation and block the effects of circulating catechoamines. In patients with VVS, the evidence for the use of β-blockers is however scant and of dubious quality (Parry and Kenny, 1999). In POTS patients β- blockers have been reported to be a useful treatment for individuals with β-receptor super-sensitivity, high noradrenaline levels and/or hyper-adrenergic states but their usefulness remain uncertain (Abed et al., 2012). Alpa-agonists such as midodrine work by increasing peripheral vascular resistance and reducing vascular capacitance (to cause increased venous return) and have been shown to be effective for patients with VVS (Ward et al., 1998). Nevertheless, despite the knowledge that sympathetic dysfunction is present in orthostatic intolerance, targeting sympathetic activity remains difficult and at present, optimal therapy still focuses on education, salt and volume management, physical counter-maneuvers and exercise.

### Conflict of interest statement

The authors declare that the research was conducted in the absence of any commercial or financial relationships that could be construed as a potential conflict of interest.
